# Comprehensive Review of Hybrid Collagen and Silk Fibroin for Cutaneous Wound Healing

**DOI:** 10.3390/ma13143097

**Published:** 2020-07-10

**Authors:** Ruth Naomi, Juthamas Ratanavaraporn, Mh Busra Fauzi

**Affiliations:** 1 Centre For Tissue Engineering & Regenerative Medicine, Faculty of Medicine, Universiti Kebangsaan Malaysia, Cheras, Kuala Lumpur 56000, Malaysia; ruthmanuel2104@gmail.com; 2 Department of Chemical Engineering, Faculty of Engineering, Chulalongkorn University, PhayaThai Road, Pathumwan, Bangkok 10330, Thailand; Juthamas.R@chula.ac.th

**Keywords:** hybrid, silk fibroin, collagen, cutaneous wound, in vitro, in vivo

## Abstract

The use of hybridisation strategy in biomaterials technology provides a powerful synergistic effect as a functional matrix. Silk fibroin (SF) has been widely used for drug delivery, and collagen (Col) resembles the extracellular matrix (ECM). This systematic review was performed to scrutinise the outcome of hybrid Col and SF for cutaneous wound healing. This paper reviewed the progress of related research based on in vitro and in vivo studies and the influence of the physicochemical properties of the hybrid in wound healing. The results indicated the positive outcome of hybridising Col and SF for cutaneous wound healing. The hybridisation of these biomaterials exhibits an excellent moisturising property, perfectly interconnected structure, excellent water absorption and retention capacity, an acceptable range of biodegradability, and synergistic effects in cell viability. The in vitro and in vivo studies clearly showed a promising outcome in the acceleration of cutaneous wound healing using an SF and Col hybrid scaffold. The review of this study can be used to design an appropriate hybrid scaffold for cutaneous wound healing. Therefore, this systematic review recapitulated that the hybridisation of Col and SF promoted rapid cutaneous healing through immediate wound closure and reepithelisation, with no sign of adverse events. This paper concludes on the need for further investigations of the hybrid SF and Col in the future to ensure that the hybrid biomaterials are well-suited for human skin.

## 1. Introduction

### 1.1. Biomaterial

Biomaterial, an element under the tissue engineering triad, is established from an advanced technology to be used as an alternative treatment worldwide. It is originated from any substance with or without any extra modification to ensure the complementary interaction with the human body. The common uses of various biomaterial designs are usually for therapeutics purposes, encompassing the treatment or diagnosis of any deteriorated conditions. A biomaterial can be modified to be used as artificial organs or provisional bioscaffolds to restore the damaged tissues. An excellent biomaterial should not trigger or only exhibit a minimal amount of immune reaction post-implantation to ensure successful tissue regeneration [1]. Usually, the fabricated biomaterial is modified to be biocompatible and bioresorbable with the human tissues [1,2]. As of now, most of the biomaterial is developed with the intention to stimulate cell response and tissue regeneration. The progressive modification of biomaterial has contributed to the development of skin substitutes such as Apligraft^®^, Dermagraft^®^, and stem cell therapy. The approved biomaterials have been proven to act as a template for the regeneration of skin in different applications [3]. Therefore, biomaterial has a huge impact on the global therapeutic market, especially in the tissue engineering segment.

The high demand for biomaterial has shown a gradual increase recently, particularly in the medical-based industry to support the current standard treatment or unresolved medical problems. Due to the drastic growth of the biomaterial industry, it is predicted that the global market for biomaterial will hit US$130 billion in the year 2020. This situation is correlated with an annual growth rate of 16% biomaterial usage worldwide [4]. In 2024, the global market for the biomaterial is expected to reach up to US$207 billion, and the major factor contributing to this phenomenon is the high demand for therapeutic implants.

Recently, various types of biomaterials are being developed, covering the natural- and synthetic-based biomaterials. As an example, most of the biomaterial commonly has been modified using collagen (Col), which resembles the extracellular matrix (ECM) characteristics. Meanwhile, silk fibroin (SF) is extensively used to produce composite scaffolds to improve the current biocompatibility and bioavailability. Col and SF can be used for tissue regeneration applications due to their resemblance to the native tissue architecture. Both can be incorporated in biomaterials for skin wound healing due to its unique physical and chemical properties. Col and SF have proven to be biologically active and biocompatible in human tissue upon being modified as a specific bioscaffold.

### 1.2. Cutaneous Wound

Any injury involving skin is commonly known as a cutaneous wound, with a major skin disruption resulting in the skin dysfunctionality. Thus, life-changing cosmetic damage and a high level of morbidity are a common complication of skin injury [5]. An effective way is required to address this issue before it worsens. Generally, the normal duration of a cutaneous wound healing is time-consuming due to its complexity and dynamic mechanism. The wound-healing phase can be categorised into four main stages: the hemostasis, inflammation, proliferative, and remodelling. Any interruption of the phases could delay wound closure and increase exposure to the infections. The skin contraction and cell ingrowth are very common in both minor and major injuries involving the skin tissue. Usually, an acute wound will end up in wound recovery due to the normal healing process. However, since it is an acute wound, the complication should not be ignored, because it might result in skin dysfunction and deformity, leading to significantly higher morbidity. Unfortunately, a severe and large open wound can cause the site’s exposure and make it prone to foreign material colonisation such as bacteria invasion. This situation might result in severe infection, as well as prolonged or non-healing wounds.

Globally, it has been estimated that about 37 million people are affected by chronic wounds from various factors [5]. A study proved that at least 10,000 people per million died from wounds infected by microbes [6]. Additionally, an average of $3927 is needed to treat a particular wound in the USA, and it could reach up to $9000 following an advanced treatment [7]. In sum, approximately $75 billion and $11 billion were estimated as the direct healthcare cost and indirect lost opportunity for skin wounds in the USA in the year 2013, respectively [8]. To overcome this issue, advanced biomaterial technology will be the best choice due to its natural property, biocompatibility characteristics, and minimum cost. Therefore, this paper reviews the hybrid of Col and SF for cutaneous wound healing.

### 1.3. Collagen

Col is the most abundant, firm, fibrous, and structural protein that acts as an ECM [9] and cellular scaffolding [10], contributing to the mechanical strength of the tissue. It is an essential element to stabilise body structure and complement most of the connective tissues that represent one-third of proteins in the human body [9]. As of today, approximately 28 types of Col have been identified, and it is dominated by type I, which makes up 90% of the human skin [7,8]. This is the main reason for the primary selection of type I Col as the major composition for cellular scaffolding in tissue engineering and regenerative medicine.

**Figure 1:**
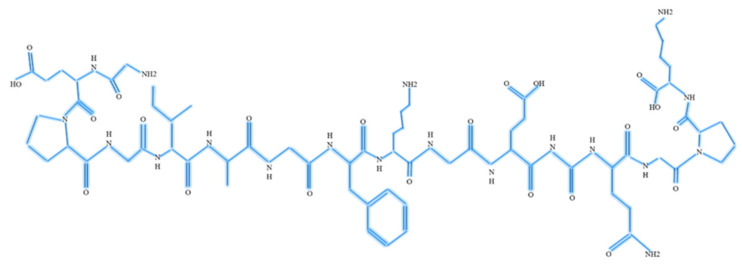
Structure of collagen.

Col appears as a triple helix of a polypeptide chain, twisted into a structure resembling a rope (Figure 1), which is naturally degraded by serine protease and collagenase [11]. The antigenicity property of Col can be reduced by removing the telopeptide from its structure [12]. The main reason is the disruption of interconnection between the cross-linked trivalent ions upon the removal of telopeptide. Consequently, the amorphous structure of Col is produced [13]. Since the cross-linked trivalent is essential for the antigenic determinant [14], the removal of telopeptide will automatically reduce the antigenicity. Col plays the main role in the formation of cellular fibrous networks to provide the optimum microenvironment, which is mostly dominated by the presence of fibroblasts [9]. Thus, Col can regulate cell proliferation, differentiation, and migration by interacting with the cell receptors in the human body. Col is one of the most extensively used natural biomaterials because it exhibits a high level of biodegradability, biocompatibility, absence of immunogenicity, and superior versatility for the fabrication of scaffolds [15]. Col can hinder the immune response by binding to glycoproteins in the platelets and leukocyte-associated immunoglobulin-like receptor-1 (LAIR-1) inhibitor. This is a major reason for Col to be known as the LAIR-1 functional ligand [16]. On the contrary, reduced dimensional and mechanical stability, enzymatic degradation, size of the fibre, the presence of impurities, the degree of cross-linking, poor elasticity [17], and high cost of obtaining pure Col type I [18] still remain as challenges for Col to be further analysed for future applications. Particularly, Col can attract fibroblasts at the wound site. This will further enhance the Col deposition at the wound bed and eventually result in the formation of new tissues through the process of autolytic debridement, the formation of blood vessels, and rapid reepithelization [19].

### 1.4. Silk Fibroin

**Figure 2:**
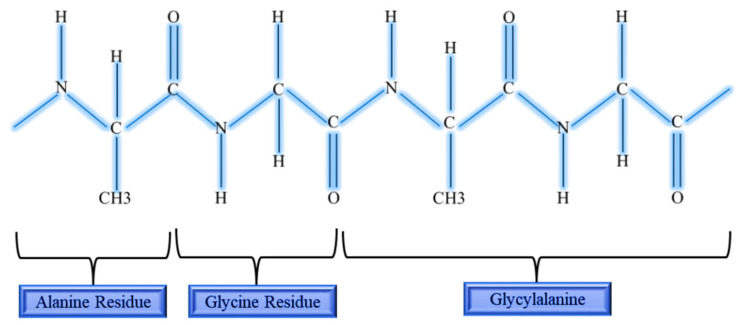
Chemical structure of silk fibroin.

SF is a complex structural protein produced by the domestic silkworm known as *Bombyx mori* which belongs to the *Golden-Yellow* species [20]. These produced silks are commonly known as fibroins due to the presence of the major components of core filament protein [21]. About 70% of fibroin comprises of fibers [22] held by water-soluble proteins known as sericin, which is a globular protein [23]. The fibroin in silk form is a glycoprotein and bonded together by a disulfide bond that consists of a polypeptide (hydrophobic molecules) [21]. These polypeptide chains are composed of helical β-sheets [24] that enhance the controlled release of drugs and a high degree of encapsulation. SF composed of both light and heavy chains. The heavy chain complements the repeated units of six different amino acid residues to the total average of 5507 amino acids [25], and the chemical structure of SF is shown in Figure 2 to represent the repeated units in heavy chain.

There are two variations of SF known as Silk I and Silk II, differentiated by the modification of the crystalline structure in the solid state [26]. Fibroin exhibits excellent tensile strength, immune tolerance, flexibility [24], biodegradability [27], high porosity, excellent compatibility with cytokines [28], and the ability to provide an antithrombotic surface [26]. Due to this unique property, SF has been used as a suture and repair material for wound closure for more than 100 years [27]. Nevertheless, this unique structure can be easily modified through a chemical reaction that alters the surface property according to the medical needs. This phenomenon makes SF superior to other available biomaterials such as polylactic acid (PLA), polyglycolic acid (PGA), and Col; in turn, this makes SF the primary choice to be used as a bioscaffold in tissue engineering. Meanwhile, the hybridisation of SF with other biomaterials has shown a promising positive result in cell adhesions [28]. The ability to alter the secondary structure of SF, specifically the biodegradation characteristics, enables it to release bioactive molecules from SF. Consequently, the release of growth factors (GF), antioxidant molecules, and antibiotics can be modulated [29]. In turn, SF is proven to support any modification intervention that supports the therapeutic applications. The GF can further stimulate the proliferation of fibroblasts and keratinocytes, favouring the environment for the deposition of Col and reepithelisation, and thereby accelerating rapid healing at the injury site [29]. Figure 2 shows the chemical structure of SF.

### 1.5. Hybrid of Collagen and Silk Fibroin

The combination of SF and Col might improve the biological and physical characteristics of the scaffold for tissue engineering and biomedical applications. This composite exhibits high kinetics during gelation, which in turn increases the survival rate of cells in 3D cultures. The hybridisation makes tuneable stiffness possible, especially in the range of 0.1 to 20 kPa, resembling the soft and hard tissue stiffness in the human body. Besides that, a high level of resistance towards various deformation indicates good elasticity, which is a major component considered for the spreading of cells in the scaffold [30]. The flexibility in compression, twisting, and bending of the scaffold is possible without losing its integrated property [30], showing the optimum toughness and strength of the scaffold. Thus, it accelerates cell growth, viability [31], and differentiation [32] due to the increased level of porosity in the scaffold [33]. The excellent porous structure indirectly influences the swelling ratio characteristic, which in turn regulates the transfer of waste and nutrition in and out of the scaffold [34]. As a result, these characteristics assist in the biological fixation at the wound bed, contributing to the dynamic wound-healing mechanism [35].

Apart from that, the stabilisation of the Col structure and the regulation of cell-mediated contraction are possible with this hybridisation of Col and SF [31]. The designed hybrid of Col and SF bioscaffold exhibits a positive correlation for cell adhesion and proliferation rate, supporting tissue regeneration such as bone and biocompatibility towards the neighbouring tissues [36]. The hybridisation of Col and SF is an excellent bioactive scaffold for tissue regeneration purposes, and recapitulated to replace the cell-based scaffold in the near future. Briefly, the hybrid exhibits perfect thermal stability and excellent mechanical strength, making it a superior scaffold compared to other polymers [37]. Besides that, it can overcome the drawbacks such as rejection by the host immune system, the limited availability of cell sources, the long duration of scaffold degradation, and the transmission of the pathogen. Nevertheless, both Col and SF are produced in large quantities from a wide range of sources such as bovine and ovine, and silkworm over the years, respectively. Ultimately, the hybridisation of Col and SF is easily translated into clinical applications after the serial requirements have been accomplished [38].

## 2. Results

The literature search identified potentially relevant articles upon assessing for the inclusion and exclusion criteria, which were selected to be further retrieved. This includes studies focusing on the in vitro and in vivo behaviour of hybrid Col and SF in cutaneous wound healing. The studies were categorised based on the study design, as shown in Table 1. Section 2 focuses on the characterisation of hybrid Col and SF and the physicochemical influence of the hybrid Col and SF in wound healing.

### 2.1. Characterisation of Hybrid Collagen and Silk Fibroin

**Figure 3:**
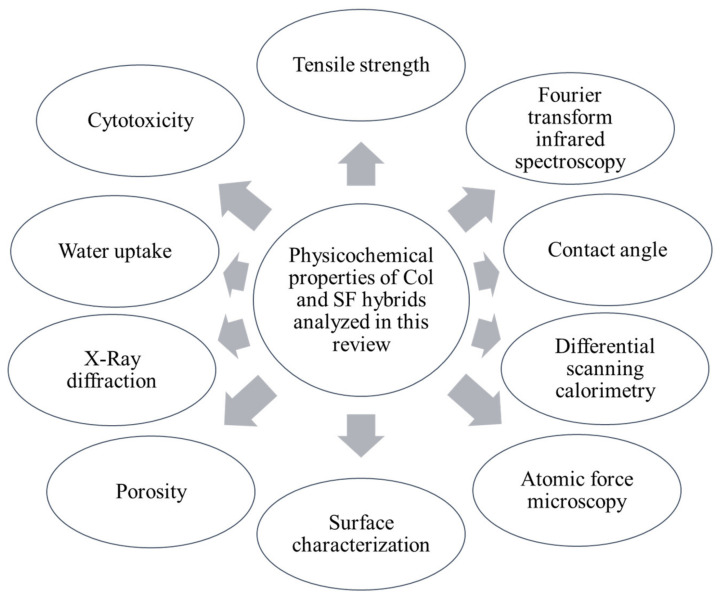
The essential physicochemical properties of collagen and silk fibroin hybrids analysed in this review.

The general characteristics of the developed scaffold greatly influenced the outcome (Figure 3). The physicochemical properties might differ according to the addition of different biomaterial compositions and cross-linking agents. Consequently, the hybridisation of Col and SF exhibited outrange physicochemical properties. These findings are summarised in Figure 3.

#### 2.1.1. Tensile Strength

Tensile strength ensures the perseverance of mechanical shear of the scaffold upon transplantation. This is vital to ensure the closure of wound at the injury site. Besides that, the persistence of hybrid scaffold is essential to ensure cell infiltration into the scaffold. To achieve this, the duration must correlate with the duration of the rebuilding of ECM. Yeelack and co-workers (2013) [39] demonstrated that the maximum load of hybridisation of Col and SF was 19.74 ± 1.15 N when the SF was incorporated with 2 mg/mL. The stress and strain of the fabricated hybridisation were 6.88 + 1.08 MPa and 6.70 + 1.38%, respectively. Significantly, the stiffness was 32.32 ± 1.59 N/mm, while the Young’s modulus was approximately 340.71 ± 30.93 MPa. The results obtained indicated that the tensile strength of the hybrid Col and SF was far superior compared to non-hybrids [39]. On the contrary, the hybrid scaffold’s elastic modulus and stress–strain decreased with the addition of SF. Meanwhile, an addition of 10% of Col decreased the elongation ratio of the hybrid scaffold [40]. Nonetheless, 1-ethyl-3-(3-dimethyl-aminopropyl-1-carbodiimide) (EDC)/N-hydroxysuccinimide (NHS) cross-linked hybrid scaffold was more rigid and less flexible, with double-fold compressive strength (124 to 234 kPa). Yet, in this case, Col concentration still influenced the value of Young’s modulus. The Young’s modulus value was higher in dry environment compared to the wet state [41].

#### 2.1.2. Fourier Transform Infrared Spectroscopy (FTIR)

The chemical characterisation of any materials can be analysed via the FTIR approach. It is essential to know the similarity of the chemical structure between the scaffold and the subject. Yeelack and co-workers (2013) [39] indicated that the particular absorbance peaks of hybridisation for amide A and B were observed at 3400–3440 cm^−1^ and 2920–2930 cm^−1^, respectively, whereas the peaks for amide I, II and III were allocated at 1640–1660 cm^−1^, 1540–1545 cm^−1^, and 1238–1250 cm^−1^, respectively [39]. A similar result was observed by Sionkowska and co-workers (2016) [41] in their study. Their results showed the peak of the α-chain at 1645, 1537, and 1241 cm^−1^ for amide I, II, and III, respectively [41]. This result was strongly supported by Kittiphattanabawon and co-workers (2010) [42], who showed that amide I, II and III were 100% together in the silk film, while the N–H represented the hydrogen bond existence in the Col [42]. Meanwhile, the hybrid scaffold cross-linked with EDC/NHS showed the same position and similar peak in IR spectra characteristics with the absence of shifts in between SF and Col. The sharp peak (β-sheet conformation) of the scaffold for amide I and II was observed at 1625 cm^−1^ and 1515 cm^−1^, respectively, whereas for α helix amide I and II, the peak was observed at 1655 cm^−1^ and 1537 cm^−1^, respectively [41].

#### 2.1.3. Contact Angle

The quantitative measure of the material wettability can be performed through contact angle assessment. This is important to quantify the hydrophilic and hydrophobic property of a scaffold. For this hybrid, the hydrophobic surface was measured >120°, while the hydrophilic was measured <120°. The results indicated that the presence of the hydrogen bond in Col contributed to the hydrophobic property of the scaffold [39]. For instance, if pure SF showed a contact angle of 90°, the addition of 10–20% of Col would drastically reduce the contact angle from 90 ± 2° to 52.5 ± 1° and 45 ± 1°, respectively. This ensures good adhesion of the cell by enhancing the hydrophilic property of the scaffold [43]. The presence of cross-linkers does not affect the changes in contact angle.

#### 2.1.4. Differential Scanning Calorimetry (DSC)

DSC indicates the thermodynamic changes that happen when the scaffold is heated or cooled down. Yeelack and co-workers (2013) [39] obtained water peaks at 80–88 °C for the hybrid. These changes in the peak temperature were noticed when the Col concentration was increased. Thus, it indicated that the Col concentration was directly proportional to the increased temperature. Meanwhile, the endothermal peak was seen at the range of 286–290 °C when the water-bound was removed. This hybrid endothermal peak was low compared to the native Col. Therefore, the presence of a crystalline structure of fibroin has been reduced with the addition of Col, clearly indicating that the combination of Col and SF demonstrated a gradually decreasing trend in the thermal stability of the silk proteins [39]. Meanwhile, Lu and co-workers (2008) [44] reported that a hybrid scaffold showed an endothermic peak at <100 °C due to the moisture microenvironment. The presence of an endothermic peak at >280 °C was due to the thermal decomposition of the non-orientated β sheet of SF. The presence of the β sheet of SF in the hybrid scaffold further enhances the stability of the hybrid scaffold [44]. A cross-linking hybrid scaffold with EDC decreased the thermal stability of the scaffold. The range was measured at 270.2 °C, which was inferior compared to the thermal point measured for SF and Col alone [45].

#### 2.1.5. Atomic Force Microscopy (AFM)

AFM measures the surface characteristics of the porous scaffold; thus, we can expect the outcome of the biological interaction of cells during an in vitro testing, assuring whether the modified scaffold is suitable to be implanted in the skin. A study performed by Yeelack and co-workers (2013) [39] showed a homogenous distribution with a smooth surface for silk. The presence of fibrils with a topographical variation was observed on the silk film, representing the hybridisation of Col and SF [39]. On the contrary, the study done by Lv and co-workers (2007) indicated that cross-linking the scaffold mixture of SF and Col with 1-ethyl-3-(3-dimethylaminoprophy) carbodiimidehydrochloride (EDC) further increased the interaction between SF and Col [45]. Consequently, it created a microenvironment that further enhanced the biological interaction of cells towards the hybrid scaffold. Increasing the density of the cross-linker was proven to enhance the mobility and induce the self-assembly of SF and Col because more and more α helix of SF were transformed into β sheet conformation [45].

#### 2.1.6. Surface Structure of Hybrid Col and SF

The surface of the hybrid showed a fibrous structure with nanofibrils. The fibrillar component on the surface indicated the SF by the appearance of homogenous pattern. However, the amount of Col concentration in the hybrid affects the surface structure appearance [39]. For instance, Yeelack and co-workers (2013) [39] in their study indicated that 0.25 mg/mL of Col incorporated with SF showed a low strand of fibril with a globular structure. In 0.5 mg/mL concentration of Col, the loosely packed parallel orientation of Col molecules was visible on the surface of the hybrid. Meanwhile, 1.0 and 2.0 mg/mL of Col concentration showed a compact structure with highly orientated Col fibrils on the surface of the hybrid [39]. Grabska-Zielińska and co-workers (2020) [46] demonstrated that the hybrid scaffold that had been cross-linked with 5% of dialdehyde starch (DAS) showed more organised structures compared to the non-cross-linked scaffold. This study was further supported by Sionkowska and co-workers (2016) [41]. They noticed a sheet-like structure with large pores in non-cross-linked scaffold, while the hybrid scaffold cross-linked with EDC/NHS showed uniform arrangement [41].

#### 2.1.7. Porosity

Optimum porosity ensures good cell viability, thereby enhancing the proliferation and growth of cells in the scaffold. A study completed by Sionkowska and co-workers (2016) [41] unraveled a highest value of porosity around 84% in the hybrid scaffold. His study proved that the increment of Col concentration was inversely proportionate to the scaffold porosity [41]. Besides that, Lu and co-workers (2009) [47] presented that scaffold porosity had been facilitated by the addition of Col composition. The hybrid scaffold revealed a pore size of 127 μm encompassing >97.7% of overall structure [47]. Meanwhile, the hybrid scaffold that had been cross-linked with DAS gradually reduced the porosity, which increased the density of the scaffold. For instance, the mixture of 89 ± 4.0% DAS cross-linker to the hybrid scaffold reduced the porosity from 97 ± 1.0 % to 91 ± 0.9 % and increased the density from 14.8 ± 2.1 mg/cm^3^ to 17.7 ± 0.4 mg/cm^3^. The increase density of the hybrid scaffold further contributed to the increase of stiffness of the matrix [46]. The increase in matrix stiffness further enhances the cell attachment and proliferation in the scaffold [48].

#### 2.1.8. X-ray Diffraction Study (XRD)

XRD detects the structural changes in the long orders of the bioscaffold. Zhou and co-workers (2010) [40] observed a stronger peak at 20.4° while the shoulder peak at 24.5° was of the hybrid that had been treated with methanol. Meanwhile, a stronger shoulder peak was seen with the addition of Col to the hybrid bioscaffold, indicating that the addition of Col simply disrupted the SF crystalline structure, resulting in varying degrees of crystallinity [40].

#### 2.1.9. Water Uptake

Zhou and co-workers (2010) [40] stated that the addition of Col strongly influenced the water uptake capacity by the hybrid scaffold. More than 70% of water uptake was seen in the hybrid bioscaffold. Besides that, the addition of Col primarily transformed the scaffold from hydrophobic to hydrophilic from 60% to >120%. This happened due to the presence of two different types of proteins in the hybrid scaffold [40]. Meanwhile, the hybrid scaffold cross-linked with DAS showed an increment with a swelling degree of 200% to 300%. At the same time, there was a decrease in moisture content when the 100 g of the hybrid scaffold was cross-linked with DAS from 19.27 ± 0.92 to 17.33 ± 0.30. This greatly affected the water uptake of the hybrid scaffold [46].

#### 2.1.10. Cytotoxicity

The hybrid of Col and SF exhibited a low level of cytotoxicity. This was proven by Long and co-workers (2016), who observed a rapid proliferation of human corneal epithelial cells (HCECs) upon cell seeding onto the fabricated scaffold. Over time, they noticed gradual changes in cell morphology with the round-shaped cell transformed into a spindle-shaped cell within 73 h. Besides that, the tremendous increment up to 200% of cell proliferation on the fifth day was reported [45]. Grabska-Zielińska and co-workers (2020) [46] described that the hybrid scaffold (SF/Col) cross-linked with DAS showed a rapid proliferation of cells when the scaffold was seeded with MG-63 cells until day 7, and less than 2% of dead cells were observed with the absence of cytotoxic events [46]. Nonetheless, the EDC cross-linked hybrid scaffold showed the positive spreading of vascular smooth muscle cells (VSMC), indicating a good biocompatibility with the cells [45].

### 2.2. Role and Physicochemical Influence of Hybrid Col and SF in Wound Healing

Human skin is made up of a multi-layered epithelium namely epidermis, dermis, and connective tissue. When an injury occurs in the skin, the ECM of the skin will be disrupted, losing its integrity [58]. Gonzalez and co-workers (2016) [59] indicated that to restore the tissue stability upon an injury, the underlying tissue needs to undergo a complex phase of healing. It commonly starts with the inflammation phase, followed by re-epithelisation, the formation of blood vessels, and remodeling [59]. Nikoloudaki and co-workers (2020) [60] reported that to cover the wounded region, keratinocytes must go through migration and proliferation stages. This is only possible with the presence of mitogen-activated protein kinases (MAP) proteins such as c-Jun N-terminal kinase (JNK) and Extracellular Signal-Regulated Kinase (ERK½). Briefly, JNK specifically influences the apoptosis, survival of cells, and epithelial sheets movements. In turn, JNK will phosphorylate another two residues known as serine 63 and serine 73. Furthermore, it will further induce the production of downstream target genes such as plasminogen activator inhibitor-I, which plays a vital role in cell migration [60]. Martínez-Mora and co-workers (2012) [22] proved that a very minimal amount, approximately 0.4% of fibroin, could accelerate the production of genes involved in cell migration. Therefore, fibroin has a direct correlation with the activation of cell migration proteins such as JNK. Meanwhile, in human keratinocytes, fibroin is involved directly in the stimulation of ERK ½ phosphorylation, thereby accelerating the re-epithelisation phase [22].

On the other hand, Di-Cosmo and co-workers (2009) [61] reported that Col, naturally being a part of the ECM, had the capacity to enhance wound healing. When there is an injury, the normal triple helix structure of the Col will be disrupted. The disrupted Col structure will bind to the fibronectin with a greater affinity [61]. According to Sevilla and co-workers (2013) [62], this might hinder the fibronectin-induced cell proliferation due to the disrupted normal interaction between Col and fibronectin [62]. Moreover, Xue and co-workers (2015) [58] stated that the normal function of Col to stimulate the platelet to release other clotting factors would be altered, thus hindering the granulation tissue formation and re-epithelisation [58]. Therefore, it slows down the normal healing phase at the site of injury and proves that adding Col at the injury site can restore and regulate the balance among matrix metalloproteinases (MMP) and proteins in ECM. These findings are essential to ensure the acceleration of the wound-healing mechanism [61].

Considering the positive effect of Col and SF, the hybridisation of these two materials further enhances wound healing, particularly with a fine modification of its physicochemical characteristics. According to Guan and co-workers (2010) [63], a hybrid scaffold with 20 to 125 μm pore size is the optimum pore size that supports skin regeneration for mammalian tissue [63]. This correlates with the study performed by Buitrago and co-workers (2018) [30], who showed that the hybrid of Col and SF contributed to stress relaxation, thereby assisting the spreading of cells through a cellular dynamic mechanism in the scaffolds [30]. Chaudhuri and co-workers (2016) [64] reported that the stress relaxation capacity influenced the ligand density for the adhesion of cells. The rapid stress relaxation contributed to the rapid integrin-based cell adhesion and enabled the fast spreading of cells in the scaffold [64]. At the same time, a hybrid of SF exhibits a high degree of positively charged arginine residues that are visible at the terminal region of carboxyl, which can easily bind to the negatively charged cell membrane. This further strengthens and accelerates the adhesion of cells with uniform morphology, resulting in active cell proliferation mainly preserved by the rigidity of the hybrid scaffold by ensuring the direction spreading of the scaffold [30].

Apart from this, Sun and co-workers (2014) [52] explained that biomaterial needed to comprise of excellent porosity capacity, water absorption characteristics, and pore size [52]. Such features are essential for cell infiltration into the bioscaffold by accelerating the diffusion of nutrition and oxygen. This in turn contributes to the mapping of architecture of tissues at the wound site. Yannas and co-workers (2015) [65] primarily described the importance of the porous structure that was easily modified to stimulate regenerative activity. An optimum diameter of the pore is essential in the scaffold development to accelerate the skin regeneration process [65], ensuring the infiltration of cells needed for wound healing in the scaffold such as inflammatory cells and fibroblasts.

Meanwhile, water-absorbing capacity ensures a moist microenvironment surrounding the scaffolds. In this scenario, Sun and co-workers (2014) [52] clarified that the hybrid scaffold exhibited water-absorption ability in the range of 1523.7 ± 186.6% that served as an optimum water-absorption capacity. This situation clearly demonstrates the accurate mass transfer of water into the scaffold, thereby ensuring the adhesion and proliferation of cells [52]. The hybrid scaffold can enhance wound healing by providing the dry wound with a suitable moisture microenvironment at the injury site. Besides that, Junker and co-workers (2013) [66] reported that the moisture perseverance at the wound site ensured the control of wound dehydration, thereby accelerating the formation of blood vessels and Col deposition. Thus, the primary contribution of the breakdown of fibrins and dead tissues at the wound area [66] made the hybrid of Col and SF an excellent biomaterial for wound healing due to its appropriate physicochemical properties observed in the in vitro studies.

At the same time, various studies have proven that SF is biocompatible to human cells. Yet, SF lacks certain domains that are needed for the binding of cells. As such, the addition of Col-I further exposes the integrin needed for the adhesion of cells in the hybrid scaffold. The ability of Col-I to transform the hybrid scaffold from the hydrophobic into hydrophilic state would accelerate the degradation property of the scaffold; hence, the addition of Col is vital. The presence of the N–H group in the Col reduces the polarity of the carboxyl group of glutamic acid present in the fibroin. According to Bai and co-workers (2008) [67], such addition results in the conversion of hydrophilic characteristics to hydrophobic property in the hybrid [67].

Zeltz and Gullberg (2016) [68] studied the composition of Col and the influence of such composition in wound healing. They reported that Col was comprised of α1β1, α2β1, α10β1, and α11β1 integrins that enhanced the remodeling of Col at the wound site. Dermal fibroblasts consisting of α11 and the deletion of fibroblasts that expressed the engrailed-1 contributed to wound healing with the absence of scarring. Since α11β1 is the main receptor for Col, it greatly affects the activated fibroblasts in tissue remodeling at the injury site [68]. The interaction of Col with SF further enhances the hydrophilicity of the bioscaffold. Briefly, SF is naturally amphiphilic, and the hydrophobic domains in this structure are disrupted by the presence of hydrophilic spacers, namely N and C terminus from the Col. Thus, the hydrophilicity increases the cell affinity to the hybrid bioscaffold, further enhancing cell adhesion and proliferation, and the rapid closure of the targeted wound.

**Figure 4:**
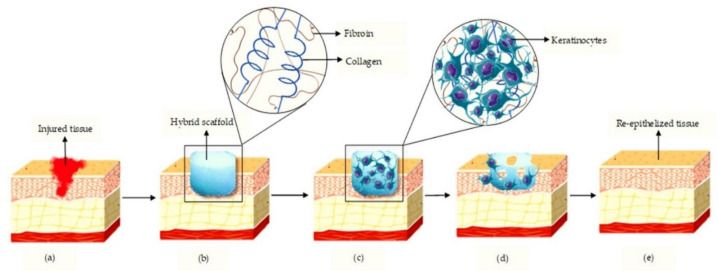
Schematic diagram showing the hybrid action of silk fibroin and collagen in cutaneous healing. (**a**) Injured tissue; (**b**) implant of hybrid scaffold at the wounded site; (**c**) Adhesion of keratinocytes to the hybrid scaffold; (**d**) Degradation of the scaffold; (**e**) Re-epithelised regenerated skin.

Besides that, the biodegradation characteristics of SF largely depend on the secondary additive. Kalaf and co-workers (2017) [69] have demonstrated that the SF biodegradation property is tuneable [69]. Thus, it indicates that a hybrid of Col and SF can be modified according to the demand for various types of cutaneous wound injury. Apart from that, Kim and co-workers (2014) [56] proved that a hybrid of Col and SF did exhibit the antioxidative properties, which were an important component in accelerating wound closure. León-López and co-workers (2019) [70] supported this by showing the degree of hydrolysis and the composition of Col powering the antioxidant property in the scaffold [70]. Antioxidant property can regulate oxidative stress at the injury site and protect from oxidative damage, thereby reducing the harm of free radicals towards the tissue. When this happens, the deleterious damage will be hindered, accelerating the rate of wound healing. Figure 4 shows the possible mechanism of action upon implanting the hybrid of SF and Col at the cutaneous wound site.

## 3. Discussion

The findings obtained from this review highlighted the beneficial effects of hybridising Col and SF in cutaneous wound healing. In the context of cutaneous wound healing, the concentration of Col incorporation with SF greatly affects the duration of wound closure as supported by the findings from the in vitro and in vivo studies in this review. The natural-based bioactive materials have been used in all the selected studies under this review [32,49,50,51,52,53,54,55,56,57] However, several studies have demonstrated additional components in the hybrid bioscaffold such as nitric oxide [54] and zinc oxide [55] for an in vitro model, and a combination with stem cells [32] in vivo that could affect the native performance of the hybrid bioscaffold containing Col and SF alone. The three-dimensional design might also influence the performance of wound healing as shown in the in vitro and in vivo models, whereby 20% of the selected articles used nanofibers and balance various designs including foam and multi-layered sheets [32,50,51,52,53,54,56,57]. All 10 articles claimed that the excellent physicochemical properties contributed to the wound-healing effect either in an in vitro or an in vivo model to heal the ischemic chronic wounds [54] and reduce scar formation [57].

In the context of biocompatibility on cellular–bioscaffold interaction towards the various types of cells, all selected articles have described the absence of any toxic effect, cell adhesion support, and cell proliferation on different days [32,49,50,51,52,53,54,55,56,57]. Mesenchymal stem cells (MSCs) are an established source of stem cells that could be used as an allogenic treatment for future use due to the limited autologous cells from a patient to expedite wound healing. MSCs have been proven viable up to 7 days in the hybrid Col and SF bioscaffold (a double layer of bovine-derived Col incorporated with a SF layer in between) [49]. The positive result on MSCs was also reported by Sun and co-workers (2014) [52] with a good distribution of MSCs inside the fabricated hybrid bioscaffold [52]. Wang and co-workers (2018) [71] also supported by correlating the promising outcome of the combination hybrid bioscaffold and MSCs, showing the naturally possessed self-renewal ability and multilineage differentiation in treating a wound model [71]. In addition to that, the use of MSC results in faster wound healing and a lower formation of scars at the injury site [32].

Besides that, the hybrid Col and SF bioscaffold has been tested using related-skin cell lines such as skin fibroblasts (L929) and NIH/3T3 cells. The hybridisation of Col and SF containing 70% of SF shows excellent biocompatibility towards fibroblast cells (L929) [51]. Hirata and co-workers (2010) [72] showed that the hybrid mimicked the skin ECM, supporting cell proliferation [72]. Similarly, an in vitro study by Bellas and co-workers (2012) [54] developed an equivalent 3D human skin through the hybridisation of Col and SF to promote cell adhesion and proliferation. Moreover, they noticed similar effects in the proliferation of NIH3T3 cells in the scaffold with regular morphology. They further proved that the hybrid scaffold did not induce any form of cytotoxic event for the future application in ischemic chronic wounds [54].

The success of the above-mentioned cell adhesion and proliferation in vitro with no sign of toxicity is closely related to the excellent selection of physicochemical properties of the fabricated hybrid Col and SF bioscaffold. Several factors have been identified to play main roles from the selected articles: the scaffold mechanical properties, pore size and porous structure, water vapor transmission rate (WVTR), degradation, surface roughness, water absorption, Fourier transformation infrared (FTIR), and crystallinity properties. The toughness, strain, and apparent modulus, which were 1.59 ± 0.39 MJ/m^3^, 50.10 ± 7.98%, and 22.79 ± 2.92 MPa, respectively, have been proven to promote the cell adhesion and growth on the hybrid Col and SF [49]. Furthermore, the additional composition of 70% of SF showed the improvement of its mechanical strength (8.7 ± 1.05 MPa), demonstrating excellent biocompatibility towards fibroblast cells [72]. The stability of the fabricated hybrid scaffold could extend the maximum cell growth up to a week and support uniform distribution in the 3D scaffold. The compressive modulus of the scaffold rapidly increased with the additional concentration of Col (3.61%), further strengthening the stiffness of the cell structure [53]. The different mechanical strength can influence the cellular behavior in three dimensions, particularly in various 3D designs such as hydrogel, sponge, thin-film, and others that promote wound healing and tissue regeneration.

Kim and co-workers (2014) [32] concluded that a 100–200 μm scaffold diameter was effective for the seeding and migration of cells. The scaffold in this study ranged from 61% to 81%, which perfectly assisted the transmigration of nutrition to the cells and exudated absorption into the scaffolds. This further enhances the proliferation of cells in the 3D scaffold [32]. The scaffolds revealed >96% of water-absorption capability, since the porosity was maintained within the range of 81% to 84%. More than 1000% of water absorbance was observed within 24 h due to the increased concentration of Col [53]. Increasing SF concentration led to large unorganised pores while 7.69% and 14.89% Col scaffold was observed to have small pores with an organised structure, indicating the perfect pore size for the proliferation of fibroblasts [53]. The infiltration of cells surrounding the scaffold (4 × 102/HP) with cell attachment in the internal surface of the scaffold being visible [52] is similar to the results acquired by Cui and co-workers (2013) [51]. Cui and co-workers (2020) [32] obtained a WVTR ranging from 52% to 64%. The stability of the scaffold was sustained up to 14 days while the degradation was observed on the 21st day [53]. The degradation property of the scaffold was identified at 330 °C and 345 °C [32].

Chemical characterisation also takes place to ensure low changes in the chemical structure and 3D conformation on hybrid Col and SF. Any obvious change on a hybrid scaffold may change the interaction on the cellular interactions, encompassing the adhesion reduction, slow proliferation, morphological change, low migration rate, and other potential adverse effects. The common characteristics including the FTIR, energy-dispersive X-ray (EDX), X-ray diffraction (XRD), and other parameters that should be taken into consideration [12]. The FTIR band results of amide I and II as stated in Table 1 indicated the transformation from random coiled conformation into a β sheet [52]. The peak of FTIR was seen at 3500–3200 cm^−1^, resembling the stretching of NH and OH present in the hybrid scaffold [53]. The FTIR of the scaffold peaks at 1627–1652 cm^–1^, presenting the amide I and amide II at approximately 1540 cm^−1^. The X-ray diffraction (XRD) showed a major diffraction peak at 21.8° on hybrid Col and SF with high crystallinity structure [57]. However, for instance, the amorphous nature of SSGH enhances the release of silk protein from the scaffold with a notable absence of cytotoxic event and antioxidative property of the hybrid scaffold [56].

From the in vivo perspective, a hybrid bioscaffold is required for further extensive study to measure the efficiency in a complex and dynamic system. The particular aspects commonly taken into consideration include the post-implantation rejection, prolonged inflammation toxic effect, and newly formed tissue stability that causes failure in tissue integration. In this review, a hybrid Col and SF bioscaffold has been proven to accelerate the wound closure and tissue regeneration either with Col and SF combination or with an additional compound to cater to the specific wound-healing phase in an in vivo model. In the inflammatory phase, the rate of healing is accelerated due to the gradually increasing expression of interleukin 6 (IL-6) and interleukin 1β (IL-1β); there is a notable increase in granulation tissue formation with a reduction in the inflammatory cells [54]. IL-6 is a mediator released from the injury site, indicating the presence of dead cells, which activate the inflammation either directly or indirectly. Meanwhile, IL-1β is an inflammatory cytokine that further stimulates the transcription factor to form and release IL-6 [73]. Luckett-Chastain and Gallucci (2009) [74] reported that IL-6 modulated the transforming growth factor beta (TGF-β) and transforming growth factor beta receptor 2 (TGF-βR2) expression in the skin [74]. TGF-β1 triggers keratinocyte migration at the wound region, thereby enhancing the deposition of the provisional matrix [75]. In this scenario, the integration of zinc oxide to the hybrid Col and SF reduces the inflammation at the injury site, thereby accelerating the wound-healing process [55].

As of today, many studies investigate MSC as an allogenic component to be applied together with bioscaffold or alone. The combination of MSC together with the Col and SF hybrid scaffold demonstrated a high formation of the matured blood vessel with >60% cell adhesion to the scaffold as observed Cui and co-workers (2020) [32], further enhancing the wound-healing mechanism [32]. Furthermore, it does regulate the immune response, thereby hindering allogenic rejection [71]. At the same time, in the late phase of the wound healing, MSC can reduce the fibroblasts’ presence to ensure the absence of Col fiber accumulation at the injury site [32]. With no doubt, this characteristic is able to accelerate the healing mechanism in the skin by promoting self-renewal of the cells at the implanted injury site.

Besides that, the hybrid Col and SF with an additional compound of S-Nitrosoglutathione, which is a bioavailable source of nitric oxide (NO), could enhance blood supply to the wound. It has the potential to dilate the blood vessels, thereby reversing back ischemic condition [54] and enhancing blood supply to the injury site. This proves that a hybrid scaffold enhances the regeneration of tissue at the skin by preserving proper microcirculation through the S-nitrosoglutathione approach [54]. This is essential particularly during the inflammatory and remodelling phase. NO further contributes to vascular permeability and the formation of blood vessels in the wound region [76]. Besides that, Kim and co-workers (2014) [56] utilise the hybrid SSGH and human Col in treating wound healing, showing fast wound healing after the implantation on a mice model [56].

The regenerated skin stability is primarily observed in the remodelling phase with a clear microstructure of epidermis and dermis layer being completely intact. The presence of keratin 10 expressions with the production of Col-I and IV enhances the tie-up of the basement membrane to the skin [50]. A study has shown that the hyperproliferation of human cells would be impossible due to the modified hybrid scaffold that comprises the normal components resembling tissue ECM [49]. Wu and co-workers (2019) [57] observed that the hybrid scaffold accelerated the normal wound-healing process with a drastic reduction in the thickness of the scar. This shows that the hybrid of SF and Col has high potential in the deposition of skin appendages [57]. Besides that, SF and Col naturally mimic the ECM of skin [77], further enhancing the bio-functional property of the hybrid scaffold and accelerating the natural healing mechanism in human skin.

From the literature search, all in vitro and in vivo studies greatly support the proliferation of cells in the hybrid Col and SF scaffold. Yet, there is a lack of evidence on human trials or clinical studies. Hence, more studies need to be done in the future to prove the safety and efficacy of this hybrid scaffold in the cutaneous wound-healing treatment. In this review, studies reporting the properties of a hybrid scaffold, the ways these properties enhance the adhesion of the cells to the scaffold, and the potential to accelerate wound healing have been discussed. The data collected throughout the studies in this review have shown that the hybrid of Col and SF naturally mimics the ECM microstructure, contributing to the acceleration of the natural healing mechanism in a cutaneous wound with an absence of any adverse or cytotoxic event.

## 4. Conclusions

The hybrid of SF and Col scaffold reveals some advantageous physicochemical properties that are impossible to be achieved with either SF or Col alone. The hybridisation of these biomaterials exhibits an excellent moisturising property, perfect interconnected structure, excellent water-absorption and retention capacity, an acceptable range of biodegradability, and synergistic effects in cell viability. The in vitro and in vivo studies have shown a promising outcome in the acceleration of cutaneous wound healing using the SF and Col hybrid scaffold. This review can be used to design an appropriate hybrid scaffold for cutaneous wound healing. Yet, further studies are still needed to be considered to predict the possible outcomes upon implanting this hybrid scaffold directly onto the human skin.

## Figures and Tables

**Table 1:** Effects of hybrid of silk fibroin and collagen in cutaneous wound healing: in vitro and in vivo analysis. FTIR: Fourier transformation infrared spectroscopy.

Author	Aim	Study Design	Follow up	Findings	Conclusion
Ghezzi and co-workers (2011) [49]	To study the hybridisation of SF and dense Col for cell proliferation	In vitro	1st, 5th and 7th day	**Physicochemical Characterisation** -FTIR peaks at 1627 cm^−1^. -Absence of alteration in structural component. -High toughness. -High tensile strength. **Cell–scaffold interaction** -Rapid cell growth of mesenchymal stem cell (MSC). -Even distribution of cell.	-The hybrid scaffold supports the viability of human skin cells. -The dermal Col resembles ECM assisting in MSC seeding in the scaffold.
Bellas and co-workers (2012) [50]	To develop a 3D human skin equivalent using silk and Col	In vitro	Varies	**Physicochemical Characterisation** -Not specified **Cell–scaffold interaction** -Polarised morphology. -Gradual increase of Col-I and Col-IV. -The level of keratin 10 peaks on day 9. -Addition of Transforming growth factor beta (TGF-β) triggers hyper proliferation.	-3D hybrid scaffold supports all type cell proliferation in human skin.
Cui and co-workers (2013) [51]	To evaluate the efficacy of Col/SF for biocompatibility of cells	In vitro	1st, 3rd and 5th day	**Physicochemical Characterisation** -Scaffold dimeter depends on the SF concentration. -The average tensile strength of the scaffold was 8.7 ± 1.05 MPa when the concentration of SF at 70%. -The amide band I appears as 1646 cm^−1^, 1647 cm^−1^, 1647 cm^−1^, 1652 cm^−1^,1652 cm^−1^ for SF concentrations of 0%, 30%, 50%, 70%, and 100%, respectively. -The amide band II appears as 1540 cm^−1^ for SF concentrations of 0%, 30%, 50%, while 1541 cm^−1^ for SF concentrations of 70% and 100%, respectively. **Cell-scaffold interaction** -Proliferation of fibroblasts (L929) was at its peak by day 5. -70% of SF concentration shows greater range of cell proliferation.	-Hybrid scaffold mimics ECM; thus, it supports cell growth and proliferation.
Sun and co-workers (2014) [52]	To test the effectiveness of SF incorporated with Col for tissue engineering	In vitro	Varies	**Physicochemical Characterisation** -The porosity was 94.6 ± 1.1%. -Highly interconnected porous with thick wall. -The water absorption capacity was 1523.7 ± 186.6%. -Young modulus data was 49.7 ± 5.0 KPa. -High compressive characteristic. **Cell–scaffold interaction** -Rapid proliferation of MSC cells. -Cell infiltration was rapid at the outer surface. -Rate of cell infiltration was at 4 × 102/HP. -Visibility of cell attachment of at the inner surface.	-Hybrid scaffold suitable for tissue engineering. -Hybrid scaffold supports cell adhesion, growth, and proliferation.
Boonrungsiman and co-workers (2017) [53]	To study the effect of hybridisation of silk-based scaffold and Col type I for skin	In vitro	1st, 3rd and 7th day	**Physicochemical Characterisation** -Addition of Col Improves porosity and stability. -Unorganised large pores with an increase of SF. -The pore size ranges from 144.09 ± 25.97 μm to 140.67 ± 38.28 μm. -Col concentration of 7.69% and 14.89%. -Intense molecular organisation at 1071 cm^−1^. -Increase concentration of Col, increase the compressive modulus. -The water-absorption capacity was exceeded up to 1000% within 30 min. -Rapid degradation at day 21. -Scaffold with 0% and 3.61% of Col concentration maintains stability up to 14 days. **Cell–scaffold interaction** -Fibroblast adhesion was at its peak in the scaffold with 50% of Col concentration. -Transformation of round-shaped fibroblasts into spindle shaped on the first day. -Small pore size enhances cell migration. -Large pore size enhances cell attachment.	-Hybrid scaffold containing 50% of Col concentration promotes a high range of cell adhesion and the proliferation of fibroblasts.
Ramadass and co-workers (2019) [54]	To study the hybrid effectiveness of type I Col peptides and nitric oxide releasing electrospun SF scaffold in treating ischemic chronic wounds	In vitro	1st, 3rd and 5th day	**Physicochemical Characterisation** -Excellent porous network and void interconnection. -Addition of Col improves hydrophilicity. -No cytotoxic effect. -Presence of antibacterial property. -Nitric oxides reaches a plateau at the 12th h. **Cell–scaffold interaction** -Excellent adherence of NIH3T3. -Regular morphology of proliferated cell. -Accelerated proliferation of cells. -Extension and spreading of cytoskeleton.	-Hybrid scaffold is proven to be biocompatible and perfect biomaterial for ischemic wound management.
Qing and co-workers (2018) [55]	To study the outcome of porous Col/SF scaffold incorporated with zinc oxide nanoparticles in wound healing	In vivo	1st, 2nd, 4th and 8th week	**Physicochemical Characterisation** -Optimum size of scaffold was at 500–600 nm. -Residual at the injury site was 3.12 ± 0.02 cm^2^, 2.75 ± 0.14 cm^2^, 2.81 ± 0.53 cm^2^, 2.34 ± 0.12 cm^2^ for the first, second, fourth and eighth hour. **Cell–scaffold interaction** -Infiltration of inflammatory cells in the control measures. -Rapid formation of granulation tissue was at the first week. -Positive expression of interleukin. -Increased deposition of mRNA expression at the wound site. -Increased deposition of granulation tissue. -Reduced inflammatory cells at the wound site. -On the 4th week, epidermal tissue exhibits a compact structure. -Rapid reepithelisation at the injury site.	-Hybrid scaffold increases the rate of healing by decreasing the inflammatory response.
Cui and co-workers (2020) [32]	To study the hybrid effectiveness of tussah SF and Col loaded with mesenchymal stem cell for wound healing.	In vivo	1st, 7th, 14th, 21st and 28th day	**Physicochemical Characterisation** -Porosity ranges from 81% to 84%. -Water absorption capacity was >96%. -WVTR ranges from 52% to 64%. -Scaffold that has been freeze-dried shows positive interconnection and porous morphology. -Degradation occurs at 330 °C and 345 °C. -Scaffold porosity increase proportional to the Col level. -Water vapor transmission rate (WVTR) inversely proportional to Col content. **Cell–scaffold interaction** -60% of cell successfully adhere to the scaffold. -The rate of cell viability increases with the increase of Col concentration.	-Hybrid scaffold promotes the maturation of blood vessels and accelerates wound healing.
Kim and co-workers (2013) [56]	To study the efficacy of human Col and silkworm gland hydrolysate (SSGH) for wound healing	In vitro and in vivo	3rd, 7th, 10th and 15th day	**Physicochemical Characterisation** -The porosity ranges from 61% to 81%. -Increased ratio of SSGH decreases the stability of the scaffold. -Greatest protein release was seen at 1:1 and 1:0 ratio of SSGH. **Cell–scaffold interaction** -Disappearance of debris. -Rapid re-epithelisation. -Rapid expansion of tissue. -Rapid migration of fibroblasts. -Absence of cytotoxicity at SSGH concentration at 0.01 g/mL to 1 g/mL.	-Hybrid scaffold enhance rapid healing from day 10 until day 15.
Wu and co-workers (2019) [57]	To study the efficiency of produced nanofibrous mat comprising of (SF)/polycaprolactone (PCL) electrospun with chitosan and Col type I in treating dermal wound and formation of scar	In vitro and In vivo	3rd, 7th and 14th day	**Physico-chemical Characterisation** -Increased mechanical strength. -Increased hydrophilic property. -Increased porosity. -Major XRD peak at 21.8°. -High crystallinity structure. -Rough surface -Good binding ability of the nanofibrous mat. **Cell–scaffold interaction** -Increased cell adhesion in cell counting kit-8 (CCK-8) assay. -Rapid cell attachment, growth, and proliferation. -Increased production of Col. -Reduced in wound-closure timing. -Reduced scar formation. -Decreased wound-healing time. -Reduced in wound exudation. -Reduced inflammation.	-Hybrid scaffold promotes blood capillary distribution. -Complete wound healing achieved at day 14 day.
